# Supplementary figures and images for: Profiling SLAs for cloud system infrastructures and user interactions
Source: PeerJ Comput Sci. 2021 May 12;7:e513. doi: 10.7717/peerj-cs.513 (PMC8157057; doi:10.7717/peerj-cs.513)

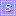

Supplement: Supplemental Information 2 — This is deployed as a Eclipse Plugin. The deployed tool with instructions to install it over Papyrus Eclipse is available at: https://www.dsi.uclm.es/cloud/modeling/uml2cloud/releases/2.1. [file peerj-cs-07-513-s002.zip › es.uclm.uml2cloud.customization/icons/SIMCAN_icon.gif]

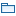

Supplement: Supplemental Information 2 — This is deployed as a Eclipse Plugin. The deployed tool with instructions to install it over Papyrus Eclipse is available at: https://www.dsi.uclm.es/cloud/modeling/uml2cloud/releases/2.1. [file peerj-cs-07-513-s002.zip › es.uclm.uml2cloud.examples/old/baseCloudSystemOld/4BEE1720.png]

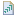

Supplement: Supplemental Information 2 — This is deployed as a Eclipse Plugin. The deployed tool with instructions to install it over Papyrus Eclipse is available at: https://www.dsi.uclm.es/cloud/modeling/uml2cloud/releases/2.1. [file peerj-cs-07-513-s002.zip › es.uclm.uml2cloud.m2t.simcan.ui/icons/default.gif]
