# Supplementary material for: Profiling SLAs for cloud system infrastructures and user interactions
Source: PeerJ Comput Sci. 2021 May 12;7:e513. doi: 10.7717/peerj-cs.513 (PMC8157057; doi:10.7717/peerj-cs.513)
Supplement: Supplemental Information 2 — This is deployed as a Eclipse Plugin. The deployed tool with instructions to install it over Papyrus Eclipse is available at: https://www.dsi.uclm.es/cloud/modeling/uml2cloud/releases/2.1. [file peerj-cs-07-513-s002.zip › es.uclm.uml2cloud.site/index.html]

UML2Cloud 2.1 Eclipse plugin update site


## Welcome to the UML2Cloud 2.1 Eclipse plugin update site.

This web page provides automatic distribution
and updates for the Eclipse plugin for UML2CLoud 2.1

### Plugin requirements

This plugin has primarily been tested with Eclipse 2019-12, but let us know if you have any problems.
The plugin runs under Java 1.8, or newer. This plugin requires at least Papyrus 5.0.0.

### Plugin installation

To install the UML2Cloud plugin:

1. In Eclipse, click on  **Help**  ->  **Install Software...** - Click  **Add**.- Enter the following:
       - **Name:**  UML2Cloud
       - **URL:**  https://dsi.uclm.es/cloud/modeling/uml2cloud/releases/2/
       and click  **OK**.- Click the checkbox next to "UML2Cloud" it to select it, and click  **Finish**.  
         **Note:** Disable **Group items by category** option.  
         **Note:** Enable **Contact all update sites during install to find required software** option.- Click **Next**.- Select the  **I accept the terms of the license agreement**  option to accept the license and click  **Next**.- The plugin is not digitally signed. Go ahead and install it anyway clicking **Ok**.- Click  **Yes**  to make Eclipse restart itself.

### Download offline version

You can download the plugin to install it offline here.
